# P-2061. Long-term Follow Up of Pfizer-BioNTech BNT162b2 mRNA Vaccinations Reveals Persistent Disparities between SARS-CoV-2 Naive and Infected Individuals in the Context of Breakthrough Infections

**DOI:** 10.1093/ofid/ofae631.2217

**Published:** 2025-01-29

**Authors:** Emelie Marklund, Anna Lundgren, Mats Bemark, Kristina Nyström, Ying Li, Aylin Yilmaz, Lars-Magnus Andersson, Magnus Gisslen

**Affiliations:** Institute of Biomedicine, Sahlgrenska Academy, University of Gothenburg, Gothenburg, Sweden, Gothenburg, Vastra Gotaland, Sweden; Institute of Biomedicine, Sahlgrenska Academy, University of Gothenburg, Gothenburg, Sweden, Gothenburg, Vastra Gotaland, Sweden; Institute of Biomedicine, Sahlgrenska Academy, University of Gothenburg, Gothenburg, Sweden, Gothenburg, Vastra Gotaland, Sweden; Institute of Biomedicine, Sahlgrenska Academy, Gothenburg, Vastra Gotaland, Sweden; University of Gothenburg, Sahlgrenska Academy, Gothenburg, Vastra Gotaland, Sweden; Institute of Biomedicine, Sahlgrenska Academy, University of Gothenburg, Gothenburg, Sweden, Gothenburg, Vastra Gotaland, Sweden; Institute of Biomedicine, Sahlgrenska Academy, University of Gothenburg, Gothenburg, Sweden, Gothenburg, Vastra Gotaland, Sweden; Biomedicine, Sahlgrenska Akademy, Gothenburg, Vastra Gotaland, Sweden

## Abstract

**Background:**

Previous studies have reported that differences in IgG and T cell responses between SARS-CoV-2 naïve and previously infected patients diminish after two vaccine doses. Yet, long-term data beyond one year and after three vaccinations is limited, with the lack of information on the impact of breakthrough infections.

**Figure:**
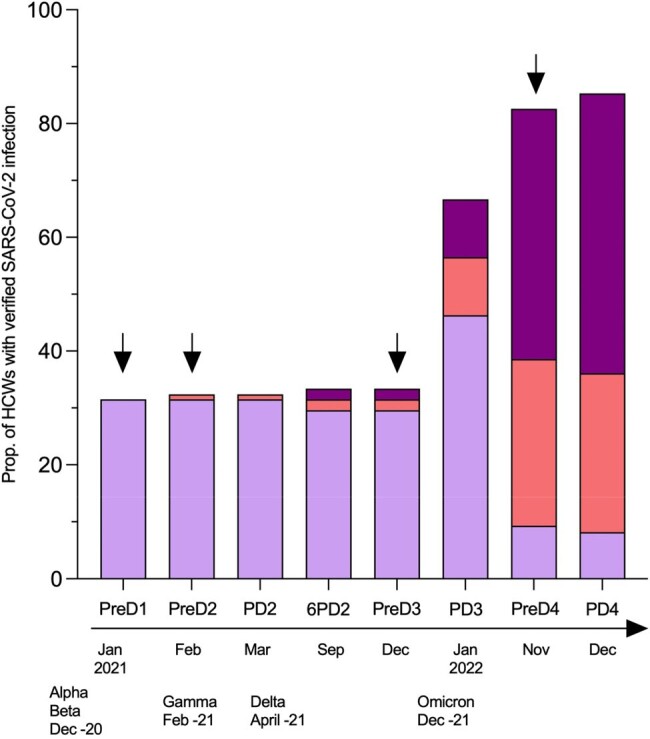

**Methods:**

108 health care workers (HCWs) from a Swedish Infectious Diseases University Clinic were included. IgG antibodies against the receptor-binding domain (RBD) and nucleocapsid (N) were measured along with cytokine-induced T cell responses (IFN-γ, IL-2, TNF-α) using an in-house assay. Samples were collected before (PreDX) and one month after (PDX) each dose of the mRNA Pfizer-BioNTech COVID-19 vaccine, from January 2021 until December 2022. Eligible HCWs underwent PCR-testing when showing SARS-CoV-2 associated symptoms. Breakthrough infection was identified by a positive PCR/antigen test or as in the N-IgG seroconversion post-vaccinations. Mean concentrations were compared between groups after adjustment for age and sex, using mixed model.

**Figure:**
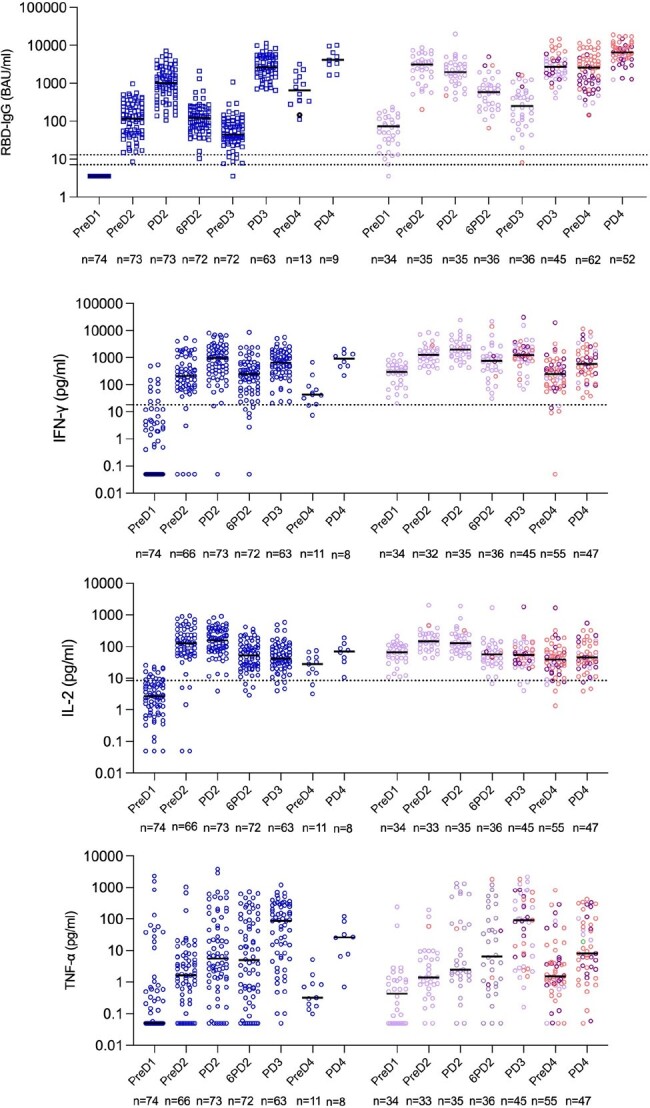

**Results:**

In total, 34 HCWs (32%) were infected prior to vaccinations. Breakthrough infections were initially few, but increased rapidly from December 2021, with 77% of the HCWs discovered with a breakthrough infection one year later (Tabel 1, Fig 1).

Infected HCWs, including those previously SARS-CoV-2 naïve with breakthrough infections, consistently had higher RBD-IgG concentrations than uninfected, except from PD3 (Figure 2). Significant differences in IFN-γ were noted at all time points except PD3 and PD4. Significant differences in IL-2 was only observed within the first month, and in TNF-α at PreD4 (Figure 2).

Those with breakthrough infections had significant higher RBD-IgG concentrations at all measured time points (PD3-PD4), with a similar pattern in IFN- γ, except from PD4.

**Figure:**
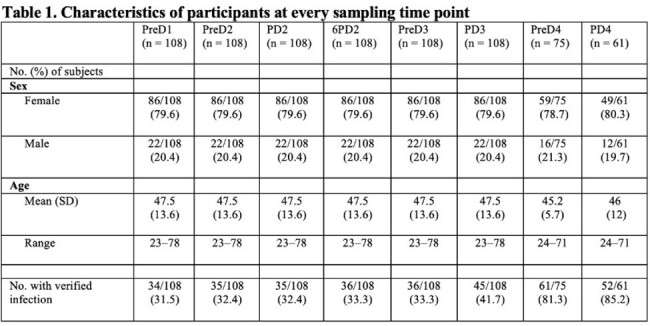

**Conclusion:**

Differences in cellular and humoral immune responses to SARS-CoV-2 between infected and naïve individuals remain significant up to 23 months post-vaccination, when considering breakthrough infections.

**Disclosures:**

All Authors: No reported disclosures

